# Exploring *Solanum tuberosum* Epoxide Hydrolase Internal Architecture by Water Molecules Tracking

**DOI:** 10.3390/biom8040143

**Published:** 2018-11-12

**Authors:** Karolina Mitusińska, Tomasz Magdziarz, Maria Bzówka, Agnieszka Stańczak, Artur Gora

**Affiliations:** 1 Tunneling Group, Biotechnology Centre, Silesian University of Technology, ul. Krzywoustego 8, 44-100 Gliwice, Poland; mitusinska@gmail.com (K.M.); tomasz.magdziarz@gmail.com (T.M.); maria.bzowka@gmail.com (M.B.); agasami@wp.pl (A.S.); 2 Faculty of Chemistry, Silesian University of Technology, ks. Marcina Strzody 9, 44-100 Gliwice, Poland

**Keywords:** epoxide hydrolases, cavities, tunnels, water trajectories, protein engineering, MD simulations, AQUA-DUCT, hot-spot

## Abstract

Several different approaches are used to describe the role of protein compartments and residues in catalysis and to identify key residues suitable for the modification of the activity or selectivity of the desired enzyme. In our research, we applied a combination of molecular dynamics simulations and a water tracking approach to describe the water accessible volume of *Solanum tuberosum* epoxide hydrolase. Using water as a molecular probe, we were able to identify small cavities linked with the active site: (i) one made up of conserved amino acids and indispensable for the proper positioning of catalytic water and (ii) two others in which modification can potentially contribute to enzyme selectivity and activity. Additionally, we identified regions suitable for *de novo* tunnel design that could also modify the catalytic properties of the enzyme. The identified hot-spots extend the list of the previously targeted residues used for modification of the regioselectivity of the enzyme. Finally, we have provided an example of a simple and elegant process for the detailed description of the network of cavities and tunnels, which can be used in the planning of enzyme modifications and can be easily adapted to the study of any other protein.

## 1. Introduction

The reaction catalysed by an enzyme is dependent on substrate availability. Whereas access of the substrates to the active site can be straightforward for enzymes with an active site exposed to the surroundings, for enzymes with buried active sites the situation seems to be much more complicated, and the substrates entry or the products exit phenomenon, occurring via tunnels, can define the rate-limiting step of the reaction [1,2]. Here an immediate question appears: ‘Why has nature complicated the structure of some biocatalysts and hid the active site inside a buried protein cavity?’ The constrained active site arrangement in 3D space, providing precise prepositioning of the amino acid functional groups, is one obvious answer [3]. Tunnels provide an access to the buried active sites and ensure more precise control of the substrates selection [4,5]. However, the possibility of controlling the reaction environment seems to be just as important as the geometrical constraints. Each enzyme provides its service immersed in the solvent that contributes to catalyst stability, activity, and selectivity [6,7,8]. Since water molecules can greatly influence the catalytic event, a variety of mechanisms regulating water access to the active site have been developed during natural evolution [9,10,11]. The division of the hydrophobic and hydrophilic compartments in the protein core can separate processes requiring distinct dielectric conditions. In enzymes with a buried active site connected with the surrounding solvent by tunnels, the water flow can be controlled much more precisely by the molecular properties of the amino acids constituting the tunnels or, in more sophisticated enzymes, by gates controlling the opening and closing of the access pathways [10,11,12]. Such control mechanisms can be much more complicated than one might expect. Various arrangements of tunnels and gates can provide the delivery of substrate or water molecules ‘on request’, thus protecting hydrophobic conditions when required and guaranteeing the water access for a hydrolytic event [13,14]. However, our understanding of solvent distribution and the flow of water molecules inside the protein core has been limited, mainly due to the lack of proper tools facilitating such analysis. We found that the exchange of water molecules occupying the active site cavity and penetrating the protein core can be investigated reasonably successfully by the use of classical molecular dynamics (MD) simulations. It is not a simple task, even though the MD simulations packages contains a variety of tools for atoms or molecules selection and the tracking of collections of molecular entities. The identification and tracking of water molecules that enter into regions important for catalysis require the screening of the positions of several thousands of single molecules along several thousands of MD steps. To fill the existing gap between tools searching for tunnels and pathways and advanced tools for accelerated water flux investigations, we have developed AQUA-DUCT, an easy-to-use tool facilitating the analysis of the behaviour of water (and if necessary other solvent molecules) penetrating any selected region in a protein [15].

An epoxide hydrolase from *Solanum tuberosum* (StEH1) is a well-studied enzyme with a buried active site and a well-defined main tunnel providing access into the catalytic triad. As an attractive target for protein engineering, it has been intensively studied using both experimental [16,17,18,19,20,21,22,23] and computational methods [18,20,23,24,25,26,27]. *Solanum tuberosum* epoxide hydrolase belongs to the α/β-hydrolase family. The core domain comprises a central eight-stranded β-sheet flanked by α-helices, with a mainly helical cap-domain (or lid-domain) positioned over the core, forming a buried active site. The active site consists of a catalytic triad, D105, D265 and H300, of the α/β domain, with two tyrosines (Y154 and Y235) from the cap domain assisting in epoxide ring opening [16,17].

Previously, several mutants have been designed to test and modify the mechanism of the reaction, substrate specificity, enantioselectivity, regioselectivity, and pH dependence (Supplementary Table S1) [16,17,19,21,22,28,29,30]. Most of the targeted residues were positioned in the binding site cavity; however, a few of them were distinct and were located on the protein surface (Supplementary Figure S1). Initial studies were dedicated to investigating the role of active site residues. Elfström and Widersten [16] mutated four catalytic candidate residues, D105, Y154, Y235, and H300, into residues with non-ionisable functional groups. They constructed seven mutants, D105A, Y154F, Y235F, Y154F/Y235F, H300A, H300N, and H300Q, with very low activity towards different TSO (trans-stilbene oxide) enantiomers and no detectable activity towards other tested epoxides [16]. Almost a decade later, Amrein et al. [30] described H300N and E35Q/H300N mutants in order to establish the principles underlying the activity and selectivity of StEH1 and proposed to expand the ‘catalytic triad’ to include E35 and H104 residues, which according to the authors’ results are indispensable for epoxide hydrolase activity. They resolved the crystal structure of the H300N mutant, which revealed a substantial perturbation of the active site.

Thomaeus et al. [19] investigated the importance and role of a putative proton wire in the StEH1 catalysis mechanism. The proton transfer path was suggested to begin from donor oxonium ions in the bulk solvent through a chain of water molecules that led to the Y154 residue in the active site. Water molecules in the main StEH tunnel were coordinated by the following residues (starting from the active site to the protein surface): Hydroxyl group of Y154 → imidazole moiety of H153 → backbone carbonyl of L266 → hydroxyl of Y149 → backbone carbonyl of H269 → backbone carbonyl of P186. Three mutant variants, Y149F, H153F and Y149F/H153F, were constructed to validate the Y149’s and H153’s integral role in this chain. The introduced single mutations resulted in a protein with a shorter estimated half-life than the wild-type (about 1 h, whereas the wild-type enzyme was stable for about 2 h and 15 min at 55 °C), whereas the double mutant showed a dramatic drop in enzyme activity with an interpolated half-life of 20 min. The single mutants H153F and Y149F also lost one and two protein-water hydrogen bonds, respectively, which destabilised the structure. Losing three hydrogen interactions in the double mutant Y149F-H153F was linked with the lowering of the protein’s half-life.

The influence of salt bridges situated between the core and cap domain and on the surface on protein stability and regioselectivity was studied by Lindberg et al. [28]. Four mutations, K179Q, E215Q, R236K and R236Q, introduced at the interface of the α/β-hydrolase fold core and the lid domains and between residues in the lid domain (residues 139–237) disrupted the salt-bridging interactions between K179-D202, E215-R41 and R236-E165 and caused increased flexibility of the protein, lower thermostability and a decrease in activity with a general trend of wild-type > K179Q > E215Q > R236K > R236Q.

The analysis of StEH1 enantioselectivity was performed in the series of studies [21,22,26,29]. Four hotspots were targeted by random mutagenesis that consisted of: A single F33 residue (hotspot A), the Y106 and L109 residues (hotspot B), V141, L145, and I155 residues (hotspot C) and I180 and F189 residues (hotspot D) [21]. Several constructed mutants were investigated in details [21,22] and the crystal structures were solved for four of them: R-C1 variant (V141K, I155V), R-C1B1 variant (W106L, L109Y, V141K, I155V), R-C1B1D33 variant (W106L, L109Y, V141K, I155V, F189L), and R-C1B1D33E6 variant (W106L, L109Y, V141K, I155V, F189L, L266G) [26,29]. All the targeted residues were situated in the binding site cavity, apart from F189, which was located behind the active site cavity. The R-C1B1 variant had an increased volume of the binding site cavity caused by the Y109 side chain’s rotation away from the binding site. The additional F189L substitution in the R-C1B1D33 variant and the L266G in the R-C1B1D33E6 variant further increased the volume of the active site pocket. The R-C1B1 variant was studied by Bauer et al. [29], who pointed out that the origin of the observed enantio- and regioselectivity is related not only to active site cavity enlargement but also to water penetration into the active site. Analysis of the F189L variant [26] also showed the importance of the F33 residue, which was found to be involved in substrate binding and might explain the degree of conservation of this residue during directed evolution (only exchanges to tyrosine were observed) [22].

Keeping in mind all the above-mentioned findings, we used AQUA-DUCT with wild-type StEH1 to investigate in detail the water flow in the vicinity of the binding site cavity and also to identify residues which may perhaps be important for the further modification of the activity and selectivity of this enzyme.

## 2. Materials and Methods

### 2.1. Protein Preparation

The crystal structure of *Solanum tuberosum* epoxide hydrolase (StEH1; PDB ID: 2CJP [17]) was downloaded from the Protein Data Bank [31]. Chain A and the ligand were removed manually from the crystal structure.

### 2.2. Molecular Dynamics Simulations

The H++ server [32] was used to protonate the structure using standard parameters and pH 6.8. Water molecules were placed using the combined method of 3D Reference Interaction Site Model (3D-RISM) [33] and the Placevent algorithm [34]. The AMBER 14 LEaP [35] was used to add counterions and immerse models in a truncated octahedral box of TIP3P water molecules and prepare the system for simulation using ff14SB force field [36]. Amber 14 [35] was used to run five repetitions of 50 ns simulations of *Solanum tuberosum* epoxide hydrolase. The minimisation procedure consisted of 2000 steps, involving 1000 steepest descent steps followed by 1000 steps of conjugate gradient energy minimisation, with decreasing constraints on the protein backbone (500, 125 and 25 kcal ·mol^−1^·Å^−2^) and a final minimisation with no constraints of conjugate gradient energy minimisation. Gradual heating was performed from 0 K to 300 K over 20 ps using a Langevin thermostat with a temperature coupling constant of 1.0 ps in a constant volume periodic box. Equilibration and production were run using the constant pressure periodic boundary conditions for 2 ns with 1 fs time step and 50 ns with a 2 fs time step, respectively. The constant temperature was maintained using the weak-coupling algorithm for 50 ns of the production simulation time, with a temperature coupling constant of 1.0 ps. Long range electrostatic interactions were modelled using the Particle Mesh Ewald method with a non-bonded cut-off of 10 Å and the SHAKE algorithm. The coordinates were saved at intervals of 1 ps.

### 2.3. Tunnel Identification

CAVER 3.02 [37] was used for tunnel calculation and clustering. The parameters used in CAVER 3.02 for the modelled protein were as follows: minimum probe radius—0.9; shell depth—4; shell radius—3; and clustering threshold—5. A portion of the 1000 tunnels was clustered by average-link clustering and used as training data. The remaining tunnels were clustered using a classifier trained on the results of the average-link clustering. The starting point was placed at the centre of mass of the catalytic active site residues’ Cα atoms, including D105, H300, D265, Y154, and Y235.

### 2.4. Water Tracking, Hot-Spot and Pocket Detection

The tracking of water molecules and the analysis of water paths, inlets and flow were performed with AQUA-DUCT software [15]. It is a universal tool that allows for the extraction, analysis and visualisation of the behaviour of solvent molecules during MD simulations.

AQUA-DUCT traces molecules that are found in the so-called object, which is usually an area of special research importance. In the present study, the object area was defined as a 4 Å sphere around the centre of geometry of five amino acids from the active site, namely D105, Y154, Y235, D265, and H300. The tracing of solvent molecules outside of the macromolecule is not particularly informative in enzyme studies. AQUA-DUCT allows setting spatial limits in which molecules are traced by allowing the user to define the so-called scope area. Here, it was defined as the interior of a convex hull of Cα atoms of the protein. Using a convex hull approach has the advantage of being very fast; however, it is quite a crude representation of the macromolecule’s shape. AQUA-DUCT allows the mitigation of this by running the Auto-Barber procedure, which additionally trims the paths of the traced molecules to the approximated surface of the macromolecule or any part of it. In order to mimic the protein surface, Auto-Barber was set to all protein atoms and van der Waals radii correction was also used.

Inlets of paths, i.e., points at which the traced molecules enters or leaves the macromolecule, were submitted to clustering routines to separate the tunnels’ exits in space, filter out outlier exits and facilitate statistical analysis.

AQUA-DUCT was also used to detect pockets in the protein core and identify amino acids surrounding cavities. This was performed by analysis of the local water molecules’ distribution in the area corresponding to all the paths of all traced molecules. The outer pocket corresponds to the maximal possible space explored by all traced molecules. AQUA-DUCT also calculates the inner pocket, which represents an area that is easily accessible by water molecules. Together with the inner pocket, AQUA-DUCT also locates high-density points or hot-spots. These are regions where water molecules are either attracted by favourable interactions with nearby amino acids or where they are trapped in hydrophobic cages. In both cases, hot-spots can mark regions of particular importance for the enzyme’s functions.

### 2.5. Evolutionary Analysis

The Multiple Sequence Alignment of 312 sequences of close homologues of soluble epoxide hydrolases deposited with the BALCONY package [38] was used for evolutionary analysis. The alignment was trimmed to span only the sequences corresponding to the analysed StEH1 structure. The phosphatase domain was removed as well as terminal leftovers. For the StEH1 crystal structure, the reference UniProt sequence was matched based on RCSB PDB information.

Using BALCONY, we analysed the MSA and mapped selected residues that made up hot-spots and cavities onto the correct positions in aligned reference UniProt sequences. The Schneider metrics were calculated for each alignment position [39].

The residues composing internal cavities identified in MD simulations of *Solanum tuberosum* soluble epoxide hydrolases were appropriately mapped with MSA, and Schneider entropy values were collected. Next, collected entropy values for all residues making up cavities were compared with the entropy values of the remainder of the protein with the non-parametric Kolmogorov-Smirnov test [40].

## 3. Results

The B chain of StEH1 crystal structure, consisting of 319 amino acids, was immersed into about 9000 water molecules, including water molecules present in the crystal model. The backbone root-mean-square deviation (RMSD) values for each simulation fluctuated near 1.2 Å (Supplementary Figure S2a). Also, the root-mean-square fluctuation (RMSF) plots for each simulation were similar. Unique high peaks stood for movements of T249 located on a P247-P256 loop between an α8 helix and a β7 sheet during the 1st simulation and for G280 located in the middle of an α10 helix during the 3rd simulation (Supplementary Figure S2b). In every simulation the two sharp peaks around G67 and N95 correspond to loops flanking α2 helix and 30-amino-acid-long peak stands for amino acids 189–219, part of the cap-domain.

### 3.1. Water Trajectories—Tunnels and Incidental Pathways

**Figure 1:**
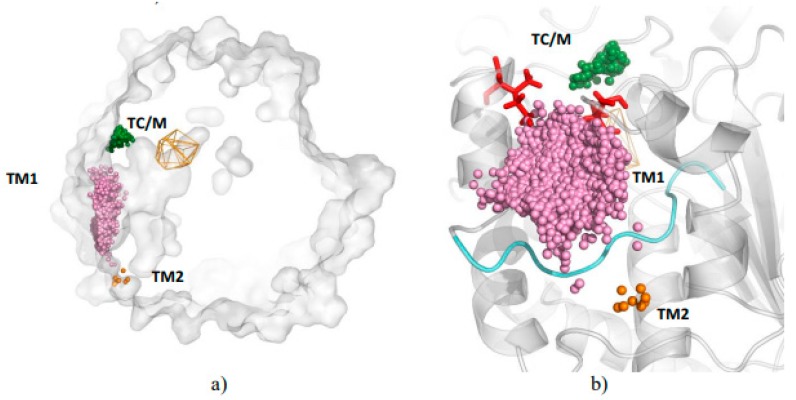
Localisation of areas of water molecule entry/egress (clusters) to active site of *Solanum tuberosum* epoxide hydrolase (StEH1): (**a**) cross-section of the protein showing network of internal cavities with large funnel providing wide access to active site (orange wire-frame); (**b**) close-up of structural features dividing the main water clusters: (i) loop (cyan) and (ii) I148 and I270 residues (red sticks). Small balls represent single inlets of water molecule entry/egress, colours correspond to identified clusters (green—TC/M, pink—TM1, orange—TM2).

**Figure 2:**
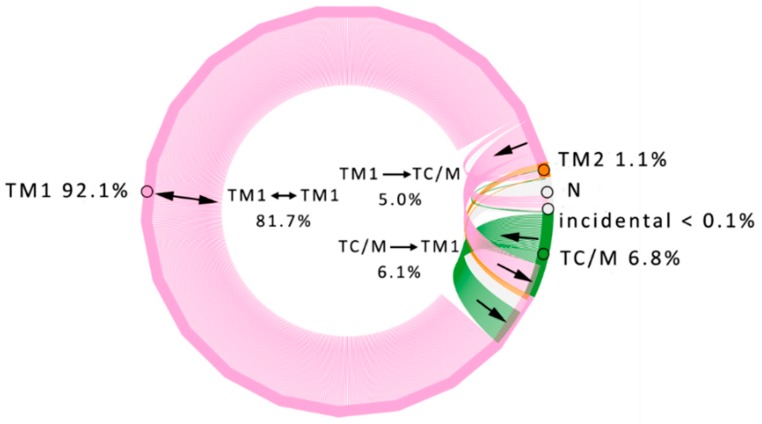
Water traffic in StEH1. The diagram shows the inter-relationships between identified inlet clusters. Clusters identified in all molecular dynamics (MD) simulations are presented as parts of the circle outline marked with consistent colour-coding: TM1—pink, TC/M—green, TM2—orange. The numbers outside the circle correspond to percentage ratios of inlets identified in particular clusters. N (grey) indicates ends of paths detected inside the protein and does not count for inlets percentage ratios. Lines inside the circle represent water paths coloured according to their entry cluster. Numbers inside the circle show ratios of water molecules travelling between annotated clusters. Please note that the largest number of water molecules enter and exit via single cluster (TM1 ↔ TM1, 81.7%). For pictures clarity, only most abundant paths (>2%, inner circle) are annotated with values and additional small arrows indicate flow direction.

The cross-section of the protein structure shows a huge cavity permanently opened to the surroundings and going deep into the protein core up to the active site (Figure 1a). It also has two branches that end close to the surface. Analysis performed by AQUA-DUCT identified that all three locations are also the main entrance/exit areas for water molecules penetrating the *Solanum tuberosum* epoxide hydrolase structure (Figure 1a). We named them as: TM1—used by the vast majority (about 90%) of the identified water molecules, permanently opened, located in a pocket between two long loops in the main domain (S129-M139 from the NC-loop, and Y183-P204 from the cap-loop, and P271); TC/M located on the border of the main and cap domains, with an entrance/exit separated from that of TM1 by I148 and I270 residues, and used for about 9% of the water molecules; and TM2 with an entrance/exit located between two loops S129-M139 and P247-P256 (approximately 1% of detected inlets, in 1st repetition, higher flexibility of the P247-P256 loop caused increase of detected inlets up to 3%) (Figure 1b and Figure 2, Supplementary Figure S2b and Table S2). Other regions with high flexible are far from detected water trajectories. The NC-loop separated the TM1 cluster from TM2. Additionally, a few outliers were detected, which identified less important, but still permeable, pathways.

The TM1 entrance corresponded to the large funnel, which provided the shortest access to the active site residues. The majority of water molecules that entered through the TM1 entrance also left by the same entrance. Only 15% of the water molecules utilising the TM1 exit came from different entrances. In contrast, in the case of the second most abundant exit, TC/M, was mostly used as a transient one, only about 11% of the water molecules entered and left by the TC/M exit. There was no preference concerning the direction of the water flow (Figure 2, Supplementary Tables S3 and S4).

We compared the identified water entry points with those recognised by the geometry-based approach. For this purpose, we used CAVER 3.02 [37] standalone version and a probe size of 0.9 Å. In each MD simulation, we were able to identify that five up to nine tunnels opened for at least 20% of the time during 50 ns MD simulations (Supplementary Table S5). In general, the main tunnel exits identified by CAVER corresponded to the egress of water molecules identified by AQUA-DUCT (Supplementary Figure S3a and Table S5). However, the main entrance, TM1, corresponded to three different tunnels identified by CAVER (Supplementary Figure S3b). This result is not surprising, keeping in mind that the CAVER is using Voronoi diagram construction for tunnels detection. Since the main cavity is very wide and asymmetric, it can accommodate several tunnels in the same frame of MD simulation, which can give an artificial picture of the tunnel network.

**Figure 3:**
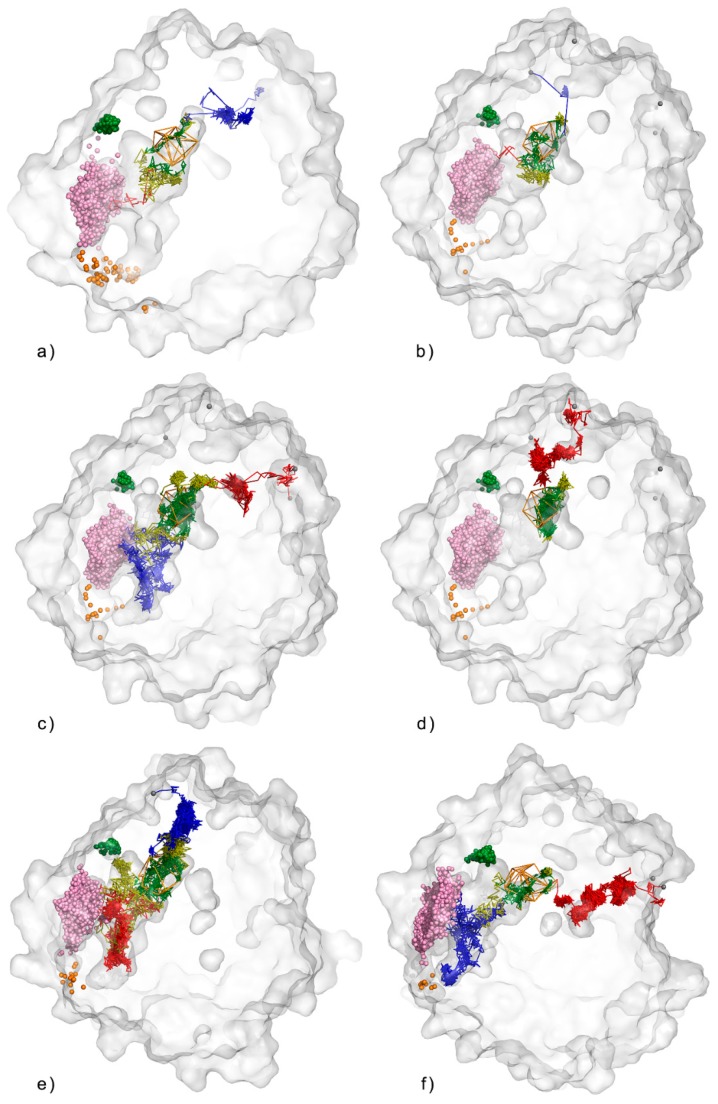
Detected water outlier trajectories in: (**a**) 1st; (**b**–**d**) 2nd; (**e**) 3rd and (**f**) 5th MD simulation. Protein shown us transparent surface, orange wireframe represents object area, Raw paths trajectories are displayed as lines. Red, green and blue lines correspond to incoming, object, and outgoing parts, respectively. Yellow lines represent part of water trajectories which leave active site and re-enter it.

**Figure 4:**
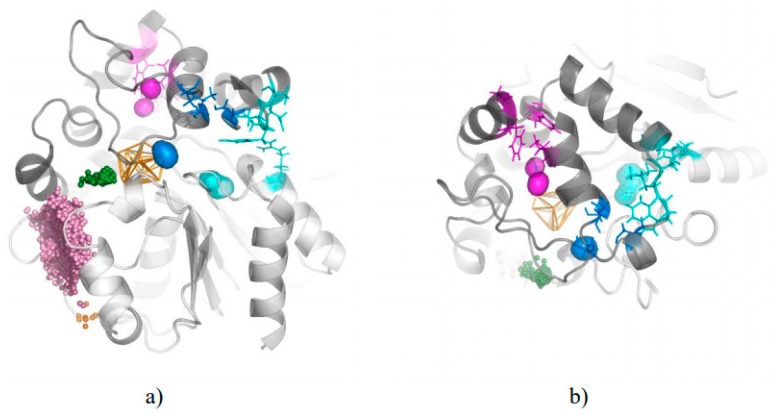
Localisation of outliers entry/exit regions. The protein side view (**a**) and top view (**b**) are shown. The core domain is shown as a light-grey cartoon, the cap domain as a dark-grey cartoon, water molecules inlets are shown as pink, green, and orange spheres for TM1, TC/M, and TM2 clusters, respectively. The cavities (interior surfaces) and residues making outliers entry/exit regions (lines) are shown in blue, cyan and magenta for I, II, and III, respectively.

Besides the main tunnels, in four out of five simulations, we detected incidental water molecules that were able to pass through the protein core end and enter/leave the protein in distinct regions (Figure 3, Supplementary Tables S4 and S6). The trajectories of such water molecules can be used to investigate alternative pathways, which possibly can be widened by rational engineering. Both the entry and egress of water outliers were detected, being localised in three regions: (i) the region I between the main and cap domains comprising R41, W210, L211, E215, and S212 residues; (ii) the region II between the residues of the cap domain loop (Y183-V205); and (iii) and the region III—the hinge region of the cap domain (E165, F168, F228, and the surrounding residues). The Figure 4 shows two views of protein with indicated outliers entry/exit regions and identified cavities described in Section 3.2. Cavities and pockets section.

Among seven detected events, four required a long time for entry or egress into the object area (>16 ns), which was caused by a water molecule being trapped in the cage of aromatic residues located between the active site and the protein surface. Others were trapped only for a short time (shorter than 1 ns) (Supplementary Table S6 and Figure S4). This observation can explain why outliers identified by AQUA-DUCT corresponded to the less populated tunnels identified by CAVER (Supplementary Figure S5). The reason may arise from the different ideas behind these tools. CAVER identifies a tunnel as a pathway that in each frame provides continuous access for a defined probe size between the starting point and the surroundings, whereas AQUA-DUCT mimics ‘real’ phenomena and detects trajectories of water which can be trapped in sub-cavities and released when particular side chains make space for further water molecules’ movement.

### 3.2. Cavities and Pockets

The analysis of the protein core revealed several small cavities, which were capable of hosting water molecules. Most of them were located close to the protein surface, but a few were deep in the protein interior. To investigate the shape of the cavities, we performed an analysis of the internal parts of water molecule trajectories (molecules penetrating the protein core, excluding incoming and outgoing parts). An analysis performed by AQUA-DUCT identified up to three cavities that were visited by water molecules passing through the active site pocket (Figure 4). Since they were connected with the active site cavity already, they could be used to reshape the binding pocket and thus expand the substrate specificity of the enzyme. Interestingly, all the cavities visited by water molecules were composed mostly of aromatic or hydrophobic residues (Table 1). They seemed to work as cages, trapping water molecules and preventing their leakage. Indeed, the identified cavities could be linked with outlier exit/entry regions (Figure 4). Cavity I was identified in all repetitions of the MD simulations, and its water-accessible volume varied significantly (from 90 to 290 Å^3^). The cavity was located between the cap and the main domain in close vicinity to the TC/M exit. The smallest water-accessible volume of cavity I was observed in the 1st simulation and was surrounded by 10 amino acids, whereas the largest one was observed in the 5th repetition and was surrounded by 24 residues (Table 1, Supplementary Figure S6a,b). Cavity II was observed in four repetitions and was positioned in the main domain. It had quite a stable water-accessible volume in all repetitions in a range of 70–100 Å^3^ and was composed of 8–13 residues; it was separated from cavity I by residues: F301 and the catalytic H300 (Table 1). The third cavity explored by water molecules, which was observed in all repetitions, was located in the hinge region, and the water-accessible volume changed from 70 to 170 Å^3^ and was surrounded by 8–15 residues (Table 1).

**Figure 5:**
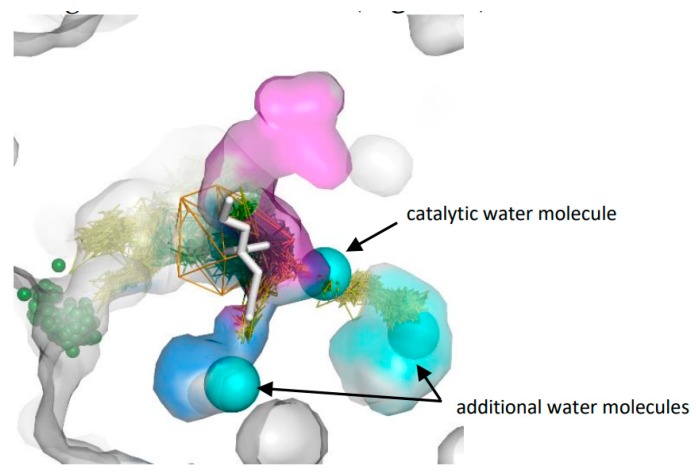
Crystal structure internal surface of chain B (grey surface), valpromide molecule (white stick) and three crystal water molecules (cyan spheres) aligned to results from AQUA-DUCT from 1st MD simulation. For picture clarity, only part of the raw trajectories and inlets from TM1 cluster (green spheres) are shown. Orange wireframe represents object area, green lines—water molecules trajectories in object area, yellow lines—water molecules trajectories which leave active site and re-enter it. Note, that cavity I (blue surface) holds the tail of valpromide, and one additional water molecule. The second tail of valpromide is located inside cavity III (magenta surface) and cavity II (cyan surface) can accommodate two water molecules (catalytic and additional).

We compared the identified cavities with the crystal structure of chain B of StEH1. Interestingly, we observed that the aliphatic regions of bound valpromide were located inside cavities I and III. Also, one of the crystallographic water molecules was found inside cavity II, ideally situated to play its role during the second step of the reaction (Figure 5) [17]. Analysis of water molecules trajectories in the rear side of the active site cavity showed that the region identified by AQUA-DUCT as cavity II could hold two water molecules, and cavities I and III could accommodate even longer substrate chains (Figure 5).

### 3.3. Trapped Water Molecules—Hot-Spot Identification

**Figure 6:**
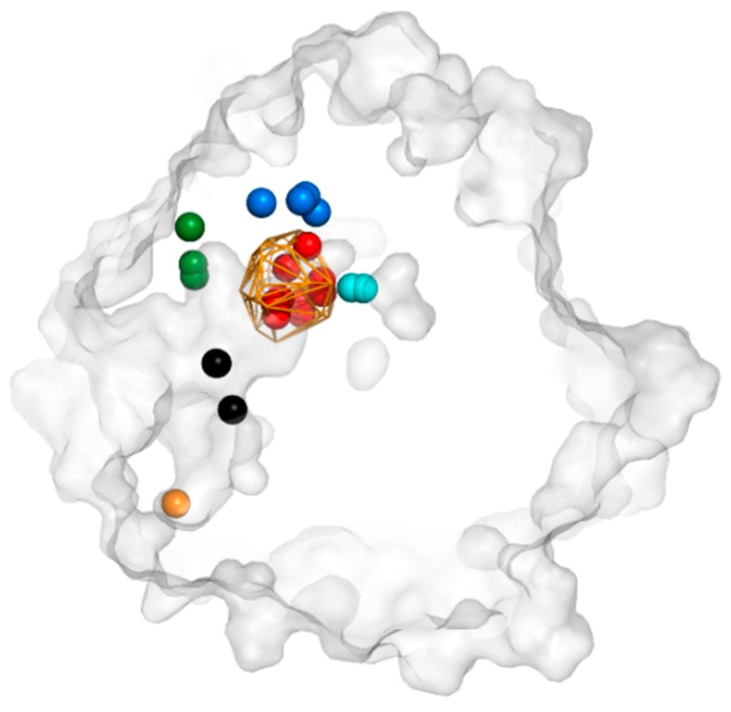
An example of identified hot-spots by AQUA-DUCT in 5th MD simulation. Protein cross-section is shown as a grey surface, object representing approximation of the active site by orange wireframe. Hot-spots are presented as balls and were identified in: active site (red), cavity I (blue), cavity II (cyan), entry to tunnel TC/M (green), entry to tunnel TM2 (orange) and in funnel interior (black).

The differences in water distribution in the protein core were applied towards the identification of residues that were able to trap water molecules. We used the local distribution of water molecules to identify such hot-spots, which might be important for water positioning or the regulation of ligand access. Interestingly, such procedures provided direct recognition of the active site itself (the largest cluster of hot-spots), detection of amino acids important for the positioning of catalytic water, residues regulating access to the protein tunnels TC/M and TM2, two cavities close to active site cavity I and cavity II, and a few positions inside the funnel (Figure 6, Supplementary Table S7).

The high density of water observed in the vicinity of the E35 residue clearly confirms its crucial role in the correct positioning of catalytic water

Our analysis suggests that one of the hot-spots surrounded by the P188, L266, and I270 residues is positioned at the entrance to the TC/M tunnel. Among them, L266 was mutated by Carlsson et al. [22]; however, it was an additional mutation in multiple variants and therefore it is difficult to interpret the role of the L266 residue. A hot-spot at the TC/M tunnel entrance was detected in all simulations. In contrast, in case of the TM2 tunnel, only in two simulations were we able to detect an increased distribution of local water molecules, and in both cases different residues were responsible for this effect (Supplementary Table S7)

Several hot-spots were identified in the funnel providing access to the active site cavity. In the majority of cases, water molecules were trapped in the vicinity of the L109, V130, H131, F132, S133, L238, N241 and W242 residues. Among them, only the L109 residue has been investigated so far, in the previously mentioned paper by Carlsson et al. [22].

The outliers provide information about water leakage, which in half of the identified cases was combined with trapping of water molecules. These phenomena were observed for cavity I, where during the 2nd simulation a water molecule was trapped for 25 ns prior to entering the active site, and during the 3rd simulation, where a water molecule was trapped for over 17 ns prior to exit. This observation was confirmed by hot-spot detection, where Y183, R184, A299, H300 and F301 were identified as key residues. A water molecule was also trapped in this cavity in the 4th and 5th simulation; however, no leakage was observed. In contrast to cavity I, water molecules that were leaking via cavity III were not trapped and left the cavity within 1 ns. Also, no hot-spot residues were detected for this cavity. Interestingly, in the 5th simulation we observed two water molecules that were trapped prior to entry to the active site for ~15 ns; however, they were trapped in a region between the surface and cavity II, which was not identified as a hot-spot.

### 3.4. Evolutionary Analysis

The Multiple Sequence Alignment (MSA) of 312 sequences of close homologues of soluble epoxide hydrolases deposited with the BALCONY (Better ALignment CONsensus analYsis) package [38] was downloaded to perform an evolutionary study. Prior to analysis, the MSA was truncated. The phosphatase domain, as well as terminal leftovers were removed. The remaining part of the MSA was used to calculate entropy values for all the amino acids. We then compared the entropy of the amino acids identified as common hot-spots.

Functional and structural perspective as well as higher number of mutations positioned on the surface suggest that variable residues are preferably located on the protein surface [41,42,43]. Indeed, the most variable residues in StEH1 are the hot-spots amino acids positioned at the surface, at the entrance to the TC/M tunnel (L266 and I270) and at the back of cavity I, separating this cavity from its surroundings (R184). Surprisingly, the majority of the residues detected in the vicinity of hot-spots areas in the interior of the protein were also annotated as variable (Supplementary Table S8). Residues that were identified as conserved created the active site (D105, H300) or played a role in catalytic water positioning (E35). H131 was identified as the only exception to this rule. It was positioned inside the funnel close to its exit, and water trapping by histidine was assisted by S133.

**Figure 7:**
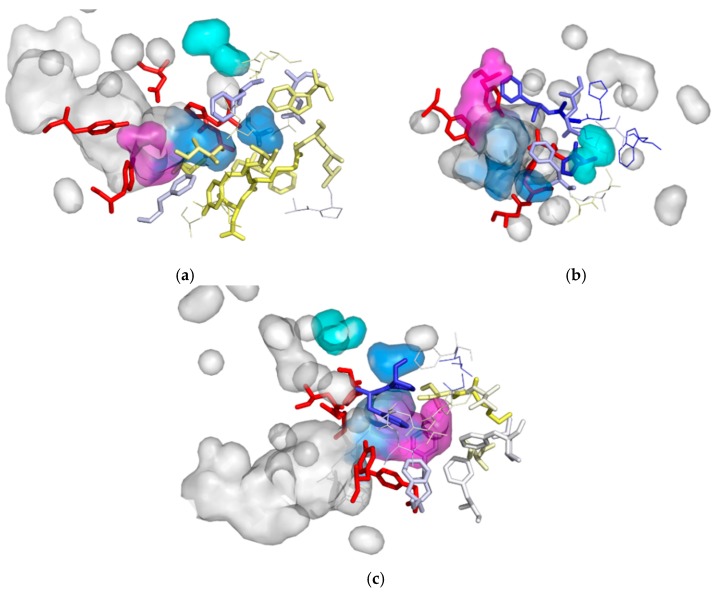
Variability of residues building cavity I (**a**), II (**b**) and III (**c**). Protein interior surface is shown in transparent grey, cavities interior surface area is shown in transparent blue (cavity I), cyan (cavity II) and magenta (cavity III), active site residues shown as red sticks, the amino acids composing cavities are coloured according to their entropy score and are shown as sticks (for frequently detected) or lines (rarely detected).

Since, according to our procedure, hot-spots are individual points in 3D space, we extended our evolutionary analysis for residues making up cavities—possibly functional compartments. The entropy scores were associated with amino acids and visualised. Close inspection suggested a large difference between the entropy scores of residues making up particular cavities (Figure 7a–c). The analytic comparisons were carried out with Kolmogorov-Smirnov tests in a similar way to that reported elsewhere [38]. According to the test results, the cavities indeed presented different evolutionary rates. Residues composing cavity II were classified as conservative ones, namely with lower entropy scores, whereas for the residues forming cavity I and III the Kolmogorov-Smirnov test failed for most of the analysed cases (α = 0.005), showing that residues forming cavity I and III evolved at a similar rate to the average rate for the whole protein.

## 4. Discussion

Depending on the planned improvement of the enzyme, protein redesign methods can vary significantly. A strategy aiming at protein stabilisation seeks to obtain better residue packing or the introduction of covalent bonds or salt bridges [44,45,46,47,48]. On the other hand, modification of the enzyme’s selectivity targets the binding site cavity or the residues composing or controlling the access pathways [1,49,50]. Recent examples show that substrate specificity regulation can be achieved by subtle changes, at a distance from the active site, which modify water or solvent accessibility [51,52,53]. Also, an entropy contribution to both binding affinity and catalysis is greatly affected by internal water positioning and dynamics, therefore mutations of residues positioning water can modify enzyme properties significantly [54,55,56]. Therefore, we applied a ligand tracking approach to examine water traffic in the interior of StEH1 and to identify potential hot-spots or regions suitable for further enzyme modification.

We assumed that our conceptually simple approach, analysis of water distribution, can identify regions where water is either attracted by favourable interactions with nearby amino acids or trapped in hydrophobic cages. In both cases, such hot-spots can mark regions of particular importance for the enzyme’s functions. Indeed, using such a procedure for hot-spot identification in the protein core, we were able to detect the most important residues and regions in the StEH1 enzyme. The active site cavity, E35 (a residue responsible for catalytic water positioning), as well as amino acids regulating the access of water via the TC/M tunnel, were easily detected. Residues surrounding hot-spots at TC/M and TM2 tunnels entry can contribute to dynamic of tunnels entrance and work as gates controlling not only solvent flow but also substrates entry or products egress. It was clearly visible during 1st repetition, where high flexibility of residues of P247-P256 loop caused an increase of water molecules traffic via TM2 tunnel. Besides these, we identified several key amino acids that can be considered for further modification of StEH1’s properties.

Using the water tracking approach, we identified and described three cavities that were directly connected with the active site and contributed to enzyme selectivity and activity. Two of them were previously reported [17] as narrow volumes that lie on the ‘inside’ of the active site cavity and in chain B of the crystal structure; they were partly filled by the two aliphatic tails of valpromide. The authors pointed out that one branch (in our study cavity I) consisted of mixed hydrophobic and polar components and eventually led out to the solvent, while the other (cavity III) was lined almost exclusively with phenylalanine side chains and was completely enclosed. In our research, we could detect both cavities in all simulations. Cavity I had the ability to expand significantly and thus protein dynamic allows to host even larger substrates than valpromide (Supplementary Figure S5a,b). In contrast to cavity I, cavity III had rather a constant size probably due to large number of π-π interactions between aromatic residues; however in one simulation, significant enlargement was observed with the cavity reaching surface residues. The AQUA-DUCT results suggested that both cavities can be permeable for water molecules; however, the events occurred over different time scales. Cavity I, which was filled by both polar and aromatic residues, provided fast transfer of water molecules, whereas ‘aromatic’ cavity III trapped water molecules prior to exit or after entry. Interestingly, passages via both cavities were reported during the entire simulation only on a few occasions. According to evolutionary analysis, both cavities (I and III) are made up of non-conserved amino acids, and thus provide an opportunity for their modification, which could run in two directions: (i) widening of a potential tunnel and thus opening an alternative pathway that could modify solvent access and thus influence substrate specificity or selectivity; or (ii) reshaping of cavities able to host larger substrates. The first strategy was successfully reported for haloalkane dehalogenase from *Sphingomonas paucimobilis* UT26 (LinB) [49], where a *de novo* tunnel was designed, and for D-amino acids oxidase, where modification of selectivity was achieved by water access modification [53]. The second strategy was previously applied to StEH1 [22,26] and other epoxide hydrolases, for example *Bacillus megaterium* epoxide hydrolase [57], where the cavity at the back was opened to accommodate larger substrates.

Interesting suggestions can arise from the merged analysis of structural and evolutionary data. The low entropy of the residues composing cavity II suggest its importance in catalysis, whereas the more variable residues creating the walls of cavities I and III suggest the possibility of their reshaping towards changed substrate specificity with potentially tolerable effect on enzyme stability. Indeed, among the residues mutated by Carlsson et al. [22], there are two, F189 and L266, that make up the walls of cavity I. The F189L and L226G mutations increased the volume of the active-site pocket without rearrangement of the position of other residues. In the case of F189L substitution, the possibility of the binding of aromatic substrates in ‘sandwiched’ mode was lost [26]. In contrast, the additional L266F mutation in the R-C1B1D33 variant had to influence the rearrangement of the surrounding amino acid side chains; however, there has been no experimental or theoretical investigation providing insight into the incorporated changes. All the tested mutants influenced the regio- and enantioselectivity of the enzyme, which proves the importance of this region in substrate positioning. The analysis of our data suggests several additional potential residues that could be used to reshape cavity I and thus further modify the enzyme’s properties.

Two amino acids detected by our approach as residues surrounding expanded cavity III have been mutated, and their impact into enzyme activity was reported in the literature. The Y154F mutant was inactive and verified the role of the hydroxyl group of the Y154 residue in the proposed mechanism of reaction [16]. The K179Q mutant was designed to examine salt-bridges’ role in enzyme stability [28]. According to our knowledge, none of the other residues surrounding cavity III have been used for StEH1 redesign so far.

As we have already pointed out, in contrast to the non-conserved cavities I and III, cavity II was found to be built by highly conserved residues. In fact, it is located in close vicinity to the 36HGXP39 sequence (where X is an aromatic residue), one of the most highly conserved motifs among EHs due to its crucial role in shaping the active site. Without the cis-peptide bond provided by this motif, the catalytic water molecule will not be correctly placed for the second step of the reaction. The alkyl-enzyme intermediate is hydrolysed by the attack of a water molecule that has been activated through proton extraction using the H300-D265 charge relay. In crystallographic structures, a crystallographic water molecule (water molecule 21 and 49 in structures A and B, respectively) was found to be ideally positioned to play this role. Its position was stabilised by the side chains of H300 and E35, as well as the backbone carbonyl oxygen of F33 [17]. The crystallographic water molecule was positioned in the entrance to cavity II. In two repetitions of our MD simulations, we detected an opening of the 2nd cavity in a manner that allowed it to accommodate two water molecules. Thus, cavity II can play a role as a reservoir of water molecules required for reaction, and protein dynamics can ensure water molecule availability after the alkyl-enzyme intermediate’s creation. As we stated in the introduction, the importance of the E35 and H104 residues was confirmed by Amrein et al., and led these authors to propose the expansion of the catalytic triad with these two residues [30]. We should also mention that in contrast to cavities I and III, we did not observe any leakage of water molecules from cavity II, which suggests that the control of the water molecule’s presence in the position required for the reaction is truly exceptional.

## 5. Conclusions

Summarising, using water molecules as a ‘molecular probe’ and employing the molecule tracking approach, we were able to investigate the structural properties of the interior of the StEH1 enzyme, providing insight into the structural compartments and hot-spots that can be targeted to reshape and modify enzyme functionality.

## Figures and Tables

**Table 1:** Amino acids making up the walls of cavities detected in particular MD simulations. Residues detected in three or more simulations in bold, residues which made up the wall of two cavities are underlined, residues reported as catalytic marked with red star, residues reported in the literature as having mutated in blue.

	Cavity I	Cavity II	Cavity III
MD1	**I180**, **T182**, **Y183**, **R184**, **F189**, D265*, **L266**, **A299**, H300*, **F301**	H31, **G32**, **F33**, **E35** *, S39, W40, **H104** *, **D105** *, G107, **H300** *, **F301**	**F33**, **P34**, **F158**, **F168**, **V176**, L177, **I180**, **F191**, F223, G227, **F228**, T229, G230, A231, V232
MD2	**I180**, **T182**, **Y183**, **R184**, **F189**, **W210**, **L266**, **A299**, H300*, **F301**, **E305**	**G32**, **F33**, P34, E35*, H104*, D105*, H300*, **F301**	**F33**, Y154*, **F158**, **F168**, **I180**, **F189**, **F191**, **I200**
MD3	**I180**, L181, **T182**, **Y183**, **R184**, **D185**, P186, A187, **F189**, **L207**, **W210**, **F264**, D265*, **L266**, **A299**, H300*, **F301**, **E305**	-	**F33**, **P34**, Y154*, **F158**, **F168**, **V176**, **K179**, **I180**, **F189**, **L197**, **I200**, **F228**
MD4	**I180**, **T182**, **Y183**, **R184**, **D185**, **F189**, A203, **L207**, S208, **W210**, **F264**, D265*, **L266**, **A299**, H300*, **F301**, **E305**	H31, **G32**, **F33**, E35*, S39, W40, H104*, D105*, L128, **H300** *, **F301**, V302, S303	**F33**, **P34**, Y154*, **F158**, **F168**, **V176**, L177, **K179**, **I180**, **F189**, **F191**, **L197**, **I200**, **F228**
MD5	K179, **I180**, L181, **T182**, **Y183**, **R184**, **D185**, P186, A187, A203, P204, **L207**, S208, **W210**, **F264**, **D265** *, **L266**, A298, **A299**, **H300** *, **F301**, V302, Q304, **E305**	**G32**, **F33**, P34, E35*, H104*, D105*, H300*, **F301**,	**F33**, Y154*, **F158**, **F168**, **V176**, **K179**, **I180**, **F189**, **F191**, **L197**, **I200**, **F228**, V232

## Supplementary Materials

The following are available online at http://www.mdpi.com/2218-273X/8/4/143/s1, Table S1: List of known StEH1 mutants, Table S2: Parameters of inlet clusters detected in StEH1 structure, Table S3: Parameters of water pathways detected in StEH1 structure, Table S4: Summary of water flow detected in all MD simulations of StEH1 structure, Table S5: Main parameters of tunnels identified by CAVER in each simulation of StEH1 structure, Table S6: Statistical data of incidental water trajectories, Table S7: Amino acids identified within 3 Å distance from detected hot-spots, Table S8: Schneider entropy scores calculated for amino acids hot-spots identified in at least 3 simulations, Figure S1: Internal water molecules trajectories merged with amino acids which were reported in the literature as selected for mutations, Figure S2: Backbone RMSD (a) and Cα RMSF (b) plots of five 50 ns simulations of StEH1 structure, Figure S3: Top 10 tunnels identified by CAVER (a) and close-up figure of main entry (b), Figure S4: Histogram presenting time required for water molecules passage through StEH1 protein, Figure S5: Superposition of identified outliers exits and corresponding tunnels from CAVER in: (a) 1st, (b) 2nd, (c) 3rd MD simulations, Figure S6: Examples of trajectories of water molecules penetrating cavity I.
