# Supplementary material for: Exploring Solanum tuberosum Epoxide Hydrolase Internal Architecture by Water Molecules Tracking
Source: Biomolecules. 2018 Nov 12;8(4):143. doi: 10.3390/biom8040143 (PMC6315908; doi:10.3390/biom8040143)
Supplement: Supplementary file 1 [file biomolecules-08-00143-s001.zip › Table_S8.pdf]

Residue	Entropy score	Position of residue
E35	0.28	active site
L109	0.74	funnel
V130	0.63	funnel
H131	0.20	funnel
F132	0.56	funnel
S133	0.72	funnel
Y183	0.79	cavity I
R184	0.83	cavity I
P188	0.68	TC/M
L238	0.73	funnel
N241	0.73	funnel
W242	0.66	funnel
L266	0.83	TC/M
I270	0.85	TC/M
A299	0.48	cavity I
H300	0.13	active site
F301	0.35	cavity I
