# Supplementary material for: Exploring Solanum tuberosum Epoxide Hydrolase Internal Architecture by Water Molecules Tracking
Source: Biomolecules. 2018 Nov 12;8(4):143. doi: 10.3390/biom8040143 (PMC6315908; doi:10.3390/biom8040143)
Supplement: Supplementary file 1 [file biomolecules-08-00143-s001.zip › Table_S1.pdf]

Variant	Stability effect	Activity effect	Selectivity effect	Ref
D105A	loss of stability	loss of activity	none	[1,6]
Y154F		loss of activity		
Y235F		loss of activity		
Y154F/Y235F		loss of activity		
H300A or H300Q		loss of activity		
H300N		some activity for R,R-TSO		
E35Q/H300N	loss of stability	some activity for R,R-TSO	none	[6]
Y149F	loss of stability	decreased activity	decrease for R,R-TSO; comparable with WT for S,S-TSO	[2]
H153F				
Y149F/H153F				
K179Q	loss of stability	increased activity towards R-enantiomer	none	[3]
E215Q		increased activity towards R-enantiomer	loss of regioselectivity; very promiscuous	
R236K or R236Q		increased activity towards S-enantiomer	higher discrimination favouring C-1 attack	
F33V or F33Y	no data	no data	none	[4]
W106F/L109I or W106F/L109V			higher discrimination favouring S-enantiomer	
V141K			lower discrimination favouring S-enantiomer	
V141K/I155V			higher discrimination favouring R-enantiomer	
W106L/L109Y/V141K/I155V			lower discrimination favouring S-enantiomer	
V141T or V141Q				
V141T/L145I or V141T/L145T				
I180L/F189C				
I180V				
F189I or F189V				
V141Q/I155L				
I180L			none	
I180C/F189V			none	
W106Y/L109I/I180F	no data	no data	shifted preference on attack on C-1 in R-SO and on C-2 in R,R-enantiomer of *trans*-2-methylstyrene oxide	[5]
W106Y/L109I/L145R/I155Y				
W106Y/L109I				
V141K/I155V			shifted preference on attack on C-2 in S-SO and on C-2 in S,S-enantiomer of *trans*-2-methylstyrene oxide	[5,7]
W106L/L109Y/V141K/I155V			shifted preference on attack on C-1 in S-enantiomer (2,3-epoxypropyl)benzene and on C-2 in R,R-enantiomer of *trans*-2-methylstyrene oxide	[5,7,8]
W106L/L109Y/I155V				[5]
W106L/L109Y/V141K/I155V/F189L				[5,7]
W106L/L109Y/V141K/I155V/F189L/L266F			shifted preference on attack on C-1 in R-SO	
W106L/L109Y/V141K/I155V/F189L/L266G			shifted preference on attack on C-2 in R,R-enantiomer of *trans*-2-methylstyrene oxide	[5]

1. Elfström L.T., Widersten M. 2005 Catalysis of potato epoxide hydrolase, StEH1. Biochem J. 390:633-640.

2. Thomaeus A., Naworyta A., Mowbray S.L., Widersten M. 2008. Removal of distal protein–water hydrogen bonds in a plant epoxide hydrolase increases catalytic turnover but decreases thermostability. Protein Sci. 17(7): 1275–1284.

3. Lindberg D., Ahmad S., Widersten M. 2010. Mutations in salt-bridging residues at the interface of the core and lid domains of epoxide hydrolase StEH1 affect regioselectivity, protein stability and hysteresis. Archives of Biochemistry and Biophysics 495: 165–173.

4. Gurell, A., Widersten, M. 2010. Modification of Substrate Specificity Resulting in an Epoxide Hydrolase with Shifted Enantiopreference for (2,3-Epoxypropyl)benzene. ChemBioChem, 11(10), 1422–1429.

5. Carlsson Å.J., Bauer P., Ma H., Widersten M. 2012. Obtaining optical purity for product diols in enzyme-catalyzed epoxide hydrolysis: contributions from changes in both enantio- and regioselectivity. Biochemistry. 25;51(38):7627-37.

6. Amrein B.A., Bauer P., Duarte F., Janfalk Carlsson Å., Naworyta A., Mowbray S.L., Widersten M., Kamerlin S.C.L. 2015. Expanding the Catalytic Triad in Epoxide Hydrolases and Related Enzymes. ACS Catal., 5 (10): 5702–5713.

7. Janfalk Carlsson Å., Bauer P., Dobritzsch D., Nilsson M., Kamerlin S.C.L., Widersten M. 2016. Laboratory-Evolved Enzymes Provide Snapshots of the Development of Enantioconvergence in Enzyme-Catalyzed Epoxide Hydrolysis. ChemBioChem 17: 1693 – 1697.

8. Bauer P., Janfalk Carlsson Å., Amrein B. A., Dobritzsch D., Widersten M., Kamerlin S. C. L. 2016. Conformational diversity and enantioconvergence in potato epoxide hydrolase 1. Org Biomol Chem. 14(24): 5639–5651.
