# Supplementary material for: Exploring Solanum tuberosum Epoxide Hydrolase Internal Architecture by Water Molecules Tracking
Source: Biomolecules. 2018 Nov 12;8(4):143. doi: 10.3390/biom8040143 (PMC6315908; doi:10.3390/biom8040143)
Supplement: Supplementary file 1 [file biomolecules-08-00143-s001.zip › Table_S2.pdf]

ID	ALL	p(ALL)	IN	p(IN)	OUT	p(OUT)
TM1	1893 ± 158	92.1 ± 7.7	940 ± 84	47.8 ± 4.1	954 ± 84	48.4 ± 4.1
TC/M	139 ± 28	6.8 ± 1.4	76 ± 9	3.9 ± 0.4	63 ± 10	3.2 ± 0.5
TM2	22 ± 23	1.1 ± 1.1	11 ± 12	0.5 ± 0.6	11 ± 13	0.6 ± 0.7
outliers	2 ± 1	< 0.1	1 ± 2	< 0.1	1 ± 1	< 0.1
