# Supplementary material for: Exploring Solanum tuberosum Epoxide Hydrolase Internal Architecture by Water Molecules Tracking
Source: Biomolecules. 2018 Nov 12;8(4):143. doi: 10.3390/biom8040143 (PMC6315908; doi:10.3390/biom8040143)
Supplement: Supplementary file 1 [file biomolecules-08-00143-s001.zip › Table_S3.pdf]

ID	IN-OUT	p(IN-OUT)	DIF	p(DIF)	N	p(N)
TM1	858 ± 87	0.84	137 ± 32	0.13	41 ± 6	0.04
TC/M	9 ± 4	0.06	118 ± 16	0.91	3 ± 1	0.02
TM2	<1 ± 1	0.01	21 ± 24	0.97	<1 ± 1	0.02
outliers	0	0.00	2 ± 1	0.75	1 ± 1	0.25
