# Supplementary material for: Exploring Solanum tuberosum Epoxide Hydrolase Internal Architecture by Water Molecules Tracking
Source: Biomolecules. 2018 Nov 12;8(4):143. doi: 10.3390/biom8040143 (PMC6315908; doi:10.3390/biom8040143)
Supplement: Supplementary file 1 [file biomolecules-08-00143-s001.zip › Table_S4.pdf]

Beginning	End	MD1	MD2	MD3	MD4	MD5	SUM	[%]
TM1	TM1	797	931	750	907	904	4289	81.7
TM1	TC/M	61	45	45	50	60	261	5.0
TC/M	TM1	65	64	53	49	90	321	6.1
TM1	TM2	28	4	6	7	4	49	0.9
TM2	TM1	25	3	9	8	2	47	0.9
TM1	N	23	19	14	18	23	97	1.8
N	TM1	25	24	21	17	21	108	2.1
TC/M	TC/M	7	7	4	13	14	45	0.9
TC/M	TM2	4	1			1	6	0.1
TM2	TC/M	3			1		4	0.1
TC/M	N	1	2	3		3	9	0.2
N	TC/M		1		2	1	4	0.1
TM2	TM2	1					1	<0.1
TM2	N	1					1	<0.1
N	TM2			1			1	<0.1
TM1	OUT	1		1	1		3	0.1
OUT	TM1		1		2		3	0.1
N	N			1			1	<0.1
OUT	N		1		1		2	<0.1
