# Supplementary material for: Exploring Solanum tuberosum Epoxide Hydrolase Internal Architecture by Water Molecules Tracking
Source: Biomolecules. 2018 Nov 12;8(4):143. doi: 10.3390/biom8040143 (PMC6315908; doi:10.3390/biom8040143)
Supplement: Supplementary file 1 [file biomolecules-08-00143-s001.zip › Table_S5.pdf]

1st MD simulation				2nd MD simulation				3rd MD simulation				4th MD simulation				5th MD simulation			
C ID	Occ [%]	AB [Å]	MB [Å]	C ID	Occ [%]	AB [Å]	MB [Å]	C ID	Occ [%]	AB [Å]	MB [Å]	C ID	Occ [%]	AB [Å]	MB [Å]	C ID	Occ [%]	AB [Å]	MB [Å]
1	100	1.9 ± 0.4	3.1	1	100	2.0 ± 0.4	2.9	1	100	1.9 ± 0.4	2.9	1	100	1.9 ± 0.4	3.0	1	100	2.0 ± 0.4	3.0
2	86	1.7 ± 0.3	2.7	2	92	1.2 ± 0.2	1.9	2	92	1.7 ± 0.3	2.8	2	97	1.3 ± 0.2	2.0	2	85	1.2 ± 0.2	2.1
3	98	1.3 ± 0.2	2.1	3	69	1.3 ± 0.3	2.6	3	97	1.2 ± 0.2	2.0	3	70	1.3 ± 0.3	2.7	3	70	1.4 ± 0.3	2.7
4	65	1.3 ± 0.2	2.5	4	30	1.2 ± 0.2	2.0	4	47	1.2 ± 0.2	2.4	4	41	1.2 ± 0.2	2.0	4	30	1.1 ± 0.2	2.0
5	47	1.2 ± 0.2	2.5	5	27	1.0 ± 0.1	1.7	5	33	1.2 ± 0.2	2.6	6	24	1.0 ± 0.1	1.4	5	47	1.2 ± 0.2	2.5
6	21	1.0 ± 0.2	2.0					6	50	1.0 ± 0.1	1.4	7	23	1.0 ± 0.1	1.3	6	23	1.0 ± 0.1	1.5
7	28	1.0 ± 0.1	1.4					7	20	1.0 ± 0.1	1.8	8	21	1.0 ± 0.1	1.4				
												9	22	1.0 ± 0.1	1.4				
												10	20	1.0 ± 0.1	1.3				
