# Supplementary material for: Exploring Solanum tuberosum Epoxide Hydrolase Internal Architecture by Water Molecules Tracking
Source: Biomolecules. 2018 Nov 12;8(4):143. doi: 10.3390/biom8040143 (PMC6315908; doi:10.3390/biom8040143)
Supplement: Supplementary file 1 [file biomolecules-08-00143-s001.zip › Table_S6.pdf]

MD	Entry	Exit	Tunnel ID (CAVER)	Residues at outliers' exit	Outliers' exit	No of events	Visited cavity	Total [ps]	Inp [ps]	Obj [ps]	Out [ps]
1	TM1	O	12	F168, A169, V176, F228	III	1	III	636	27	273	330
2	TM1	O	11	Y190, A199	II	1	III	619	18	537	64
2	O	TM1	8	E165, F168, A169, G172, A173, E224	III	2	III	2206<br>2464	526<br>798	1138<br>958	542<br>708
2	O	N	14	K178, T182, A203, V205, S208	II	1	I	27707	25485	2222	N
3	TM1	O	16	T182, R184, A203, P204, L207, S208	II	1	I	22547	1513	3439	17595
5	O	TM1	n.i.	R41, W210, L211, S212, E215, Q304	I	1	-	17423	15226	531	1664
5	O	N	n.i.	R41, L211, S212, E215	I	1	-	19915	14666	5249	N
