# Supplementary material for: Exploring Solanum tuberosum Epoxide Hydrolase Internal Architecture by Water Molecules Tracking
Source: Biomolecules. 2018 Nov 12;8(4):143. doi: 10.3390/biom8040143 (PMC6315908; doi:10.3390/biom8040143)
Supplement: Supplementary file 1 [file biomolecules-08-00143-s001.zip › Table_S7.pdf]

Hot-spot localization	MD1	MD2	MD3	MD4	MD5
TC/M entry	H153, **P188**, **L266**, **I270**	P186, A187, **P188**, **L266**, **I270**	**P188**, **L266**, **I270**	Y149, **P188**, **I270**	Y149, P186, A187, **P188**, H269, **I270**
TM2 entry		L116, F132, W248, A251			R135, T249
cavity I		T182, **Y183**, **R184**, **A299**, **H300***, **F301**	**Y183**, **R184**, D185, F264, L266, **A299**, **H300***	**Y183**, **R184**, **A299**, **H300***, **F301**	I180, **Y183**, **R184**, D185, F189, F264, L266, **A299**, **H300***, **F301**
cavity II		F33, **E35***, D105*, H300*, F301		F33, **E35***, H300*, F301	**E35***, D105*, H230
funnel interior	**L109**, **V130**, **H131**, **F132**, **S133**, **L238**, **N241**, **W242,** V267	I78, **L109**, H113, **V130, H131**, **F132**, **S133**, N140, V141, **L238**, **N241**, **W242,** T245	**L109**, **V130,** V141, **L238**, **N241**, **W242**	**L109**, **V130**, **H131,** **S133**, R135, **N241,** T245, W248	**L109**, **V130**, **H131, F132**, **S133**
