# Supplementary figures and images for: Exploring Solanum tuberosum Epoxide Hydrolase Internal Architecture by Water Molecules Tracking
Source: Biomolecules. 2018 Nov 12;8(4):143. doi: 10.3390/biom8040143 (PMC6315908; doi:10.3390/biom8040143)

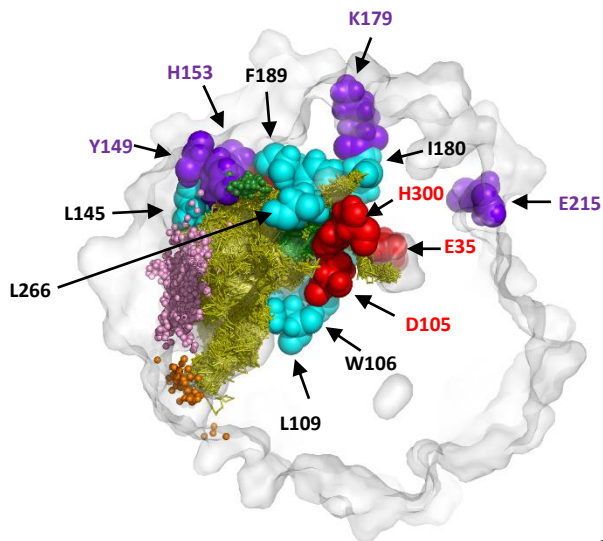

a)

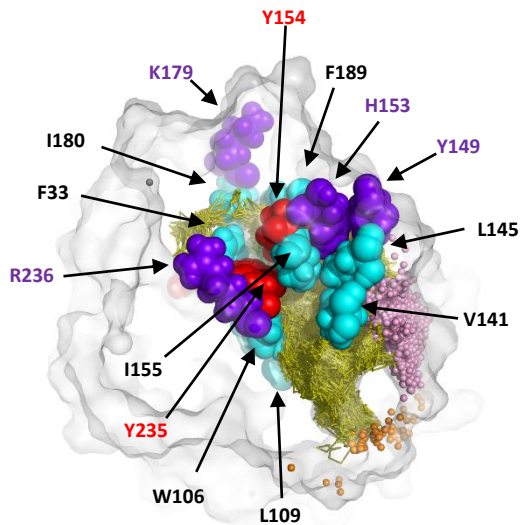

b)

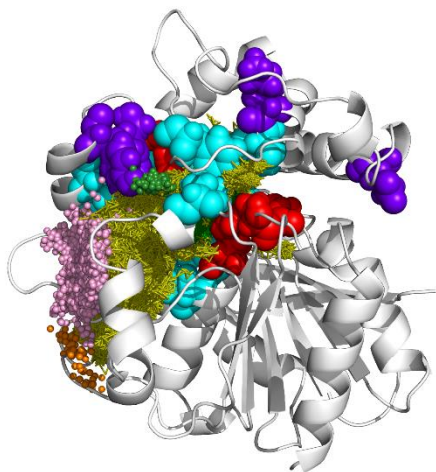

c)

Supplement: Supplementary file 1 [file biomolecules-08-00143-s001.zip › Figure_S1.pdf]

RMSD backbone

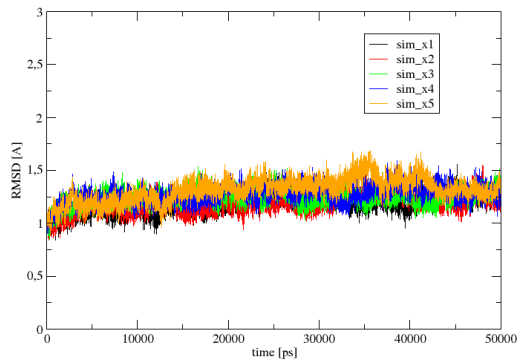

a)

RMSF CA

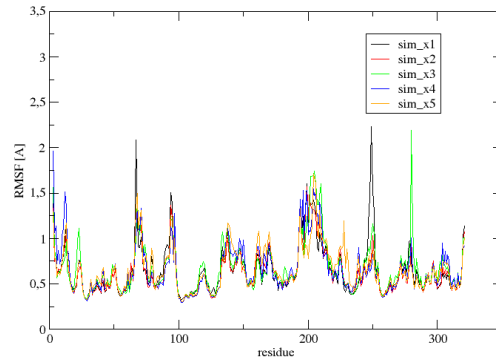

b)

Supplement: Supplementary file 1 [file biomolecules-08-00143-s001.zip › Figure_S2.pdf]

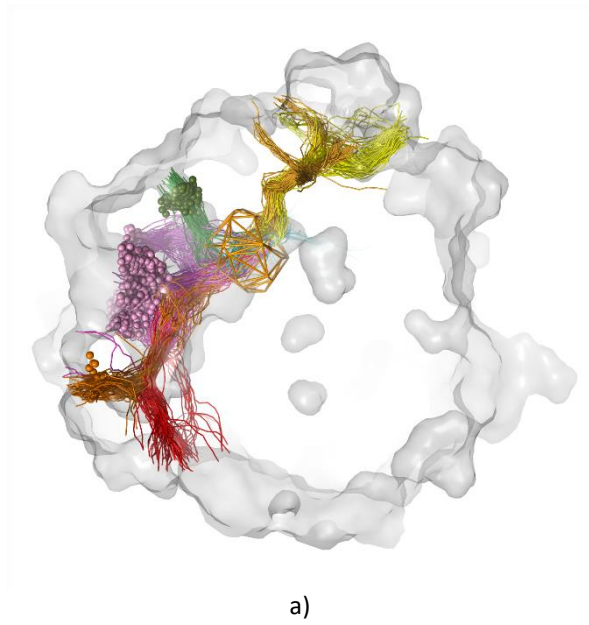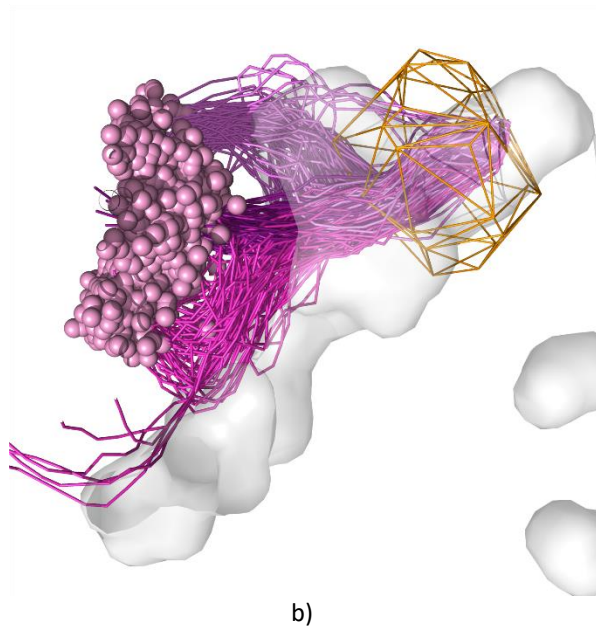

Supplement: Supplementary file 1 [file biomolecules-08-00143-s001.zip › Figure_S3.pdf]

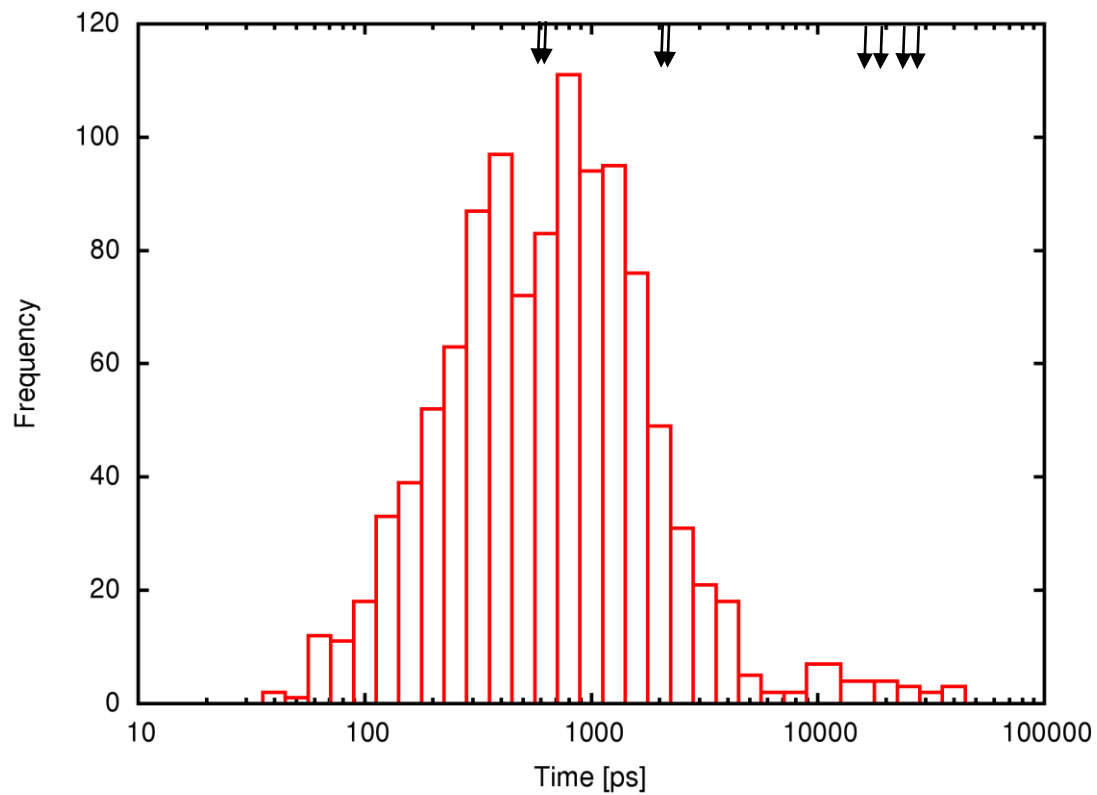

Supplement: Supplementary file 1 [file biomolecules-08-00143-s001.zip › Figure_S4.pdf]

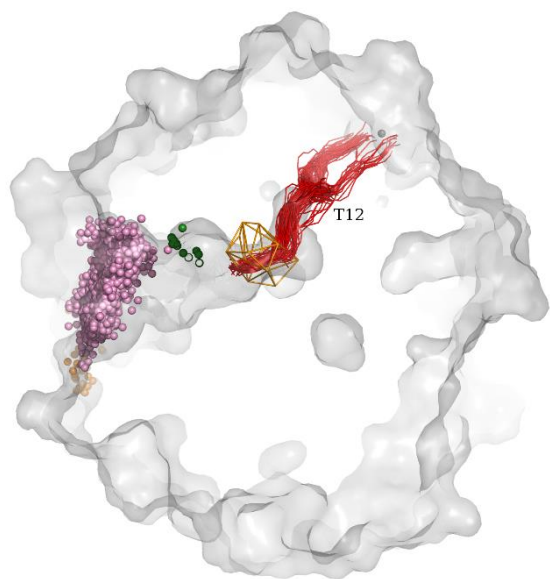

a)

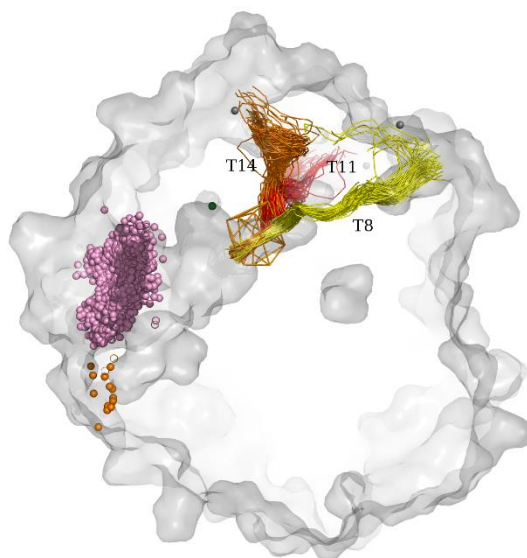

b)

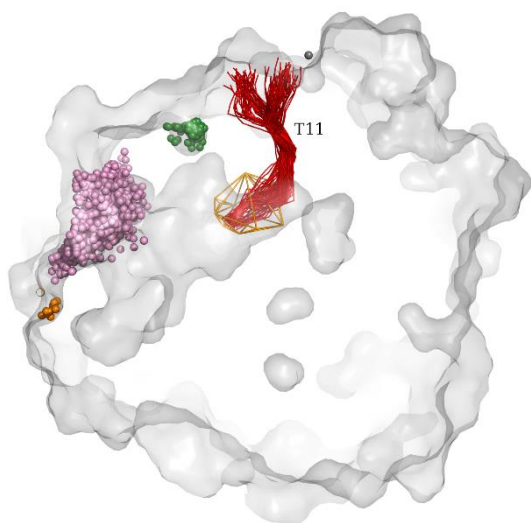

c)

Supplement: Supplementary file 1 [file biomolecules-08-00143-s001.zip › Figure_S5.pdf]

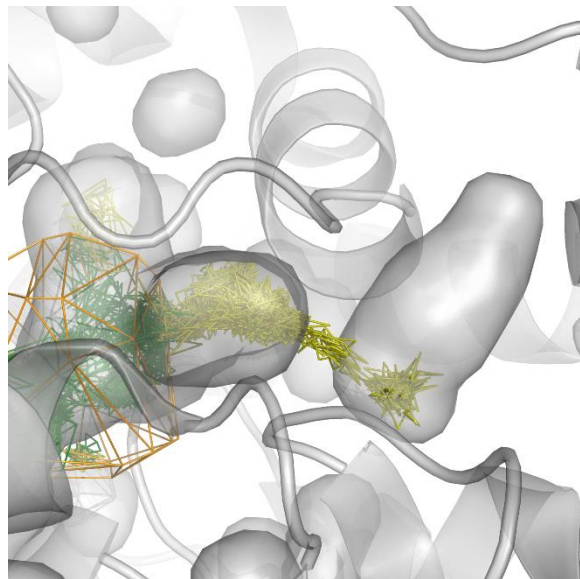

a)

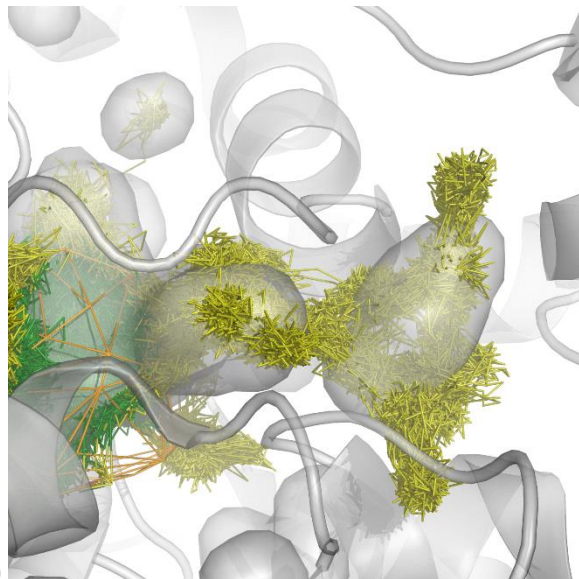

b)

Supplement: Supplementary file 1 [file biomolecules-08-00143-s001.zip › Figure_S6.pdf]
