# Supplementary material for: Exploring Solanum tuberosum Epoxide Hydrolase Internal Architecture by Water Molecules Tracking
Source: Biomolecules. 2018 Nov 12;8(4):143. doi: 10.3390/biom8040143 (PMC6315908; doi:10.3390/biom8040143)
Supplement: Supplementary file 1 [file biomolecules-08-00143-s001.zip › Figure_S1.doc]

**K179**

**Y154**

a) b)

**E215**

**Y149**

**H153**

**H300**

**D105**

**E35**

**L145**

**F189**

**I180**

**L266**

**L109**

**W106**

**K179**

**R236**

**H153**

**Y149**

**Y235**

**I180**

**F189**

**F33**

**I155**

**L145**

**V141**

**L109**

**W106**

c)
