# Supplementary material for: Exploring Solanum tuberosum Epoxide Hydrolase Internal Architecture by Water Molecules Tracking
Source: Biomolecules. 2018 Nov 12;8(4):143. doi: 10.3390/biom8040143 (PMC6315908; doi:10.3390/biom8040143)
Supplement: Supplementary file 1 [file biomolecules-08-00143-s001.zip › Supplementary captions.doc]

Supplementary captions.

Supplementary Table 1. List of known StEH1 mutants.

Supplementary Figure 1. Internal water molecules trajectories merged with amino acids which were reported in the literature as selected for mutations. StEH1 crystal structure (PDB ID: 2CJP) shown as surface (a and b) or cartoon (c) representation. Mutations of active site are shown as red, mutations altering the hydrogen bonding and electron transfer as purple and those altering enantioselectivity as cyan CPK, active site cavity as orange wireframe. Inlets of water molecule entry/egress are shown as dark-green, pink and orange balls. Raw paths trajectories are displayed as lines. Red, green and blue lines correspond to incoming, object, and outgoing parts, respectively. Yellow lines represent part of water trajectories which leave active site and re-enter it. Please note, that majority of residues mutated so far were positioned in the vicinity of the active site or close to the TC/M entry. Only few amino acids were mutated in the cap domain.

Supplementary Figure 2. Backbone RMSD (a) and Cα RMSF (b) plots of five 50 ns simulations of StEH1 structure. The black, red, green, blue and orange lines represent consecutive simulation runs.

Supplementary Table 2. Parameters of inlet clusters detected in StEH1 structure. Table shows the identified cluster ID, the total number of inlets for particular cluster (ALL), the number of incoming (IN), and outgoing (OUT) water paths. The p(ALL), p(IN), and p(OUT) show percentage ratios for inlets detected in all simulations.

Supplementary Table 3. Parameters of water pathways detected in StEH1 structure. Table shows the identified cluster ID, the number of paths of water molecules that entered and left the active site using the same inlet cluster (IN-OUT), entered or left the active site using different inlet cluster (DIF), and entered the active site and stuck or stayed in the active site and left (N). The p(IN-OUT), p(DIF), and p(N) show probability of the particular event per cluster.

Supplementary Table 4. Summary of water flow detected in all MD simulations of StEH1 structure. Table shows statistical data of all detected pathways. N indicates ends of trajectories detected inside the protein core, OUT indicates outliers.

Supplementary Table 5. Main parameters of tunnels identified by CAVER in each simulation of StEH1 structure. Corresponding clusters defined by AQUA-DUCT are indicated by colours: TM1 pink, TC/M green, TM2 orange. For each simulation, a tunnel ID (C ID), tunnel occupancy (Occ), average (AB) and maximum (MB) bottleneck are provided. Please note, that in each simulation more than one tunnel correspond to TM1 cluster. Tunnels which were identified in less than 20% of MD simulations frames were omitted for table clarity.

Supplementary Figure 3. Top 10 tunnels identified by CAVER (a) and close-up figure of main entry (b).

Supplementary Figure 4. Histogram presenting time required for water molecules passage through StEH1 protein. Arrows mark total times of passage of detected incidental water trajectories (outliers) from Supplementary Table 5.

Supplementary Figure 5. Superposition of identified outliers exits and corresponding tunnels from CAVER in: a) 1st, b) 2nd, c) 3rd MD simulations. There was no outliers in 4th simulation and we were unable to match outliers in 5th simulation with any tunnels detected by CAVER. Numbers correspond to tunnels ID from CAVER.

Supplementary Table 6. Statistical data of incidental water trajectories. Total, Inp, Obj, and Out correspond to time calculated for water molecules during their passage inside the protein scope, the incoming part, the object part, and the outgoing part, respectively.

Supplementary Table 7. Amino acids identified within 3 Å distance from detected hot-spots. Table shows residues detected in at least 3 simulations (bold), residues which build wall of two cavities (underlined), residues reported as catalytic (marked with red star), mutated residues reported in literature (blue). Please note, that identified amino acids in particular region (e.g., in the funnel interior) can represent several hot spots distinct in space.

Supplementary Table 8. Schneider entropy scores calculated for amino acids hot-spots identified in at least 3 simulations.

Supplementary Figure 6. Examples of trajectories of water molecules penetrating cavity I. Object (orange wireframe), trajectories within object (green lines), trajectories inside the protein scope but not inside the object (yellow lines), protein (cartoon), interior cavity from crystal structure (surface) are shown for 2nd simulation (a), and 4th simulation (b). Please note, that area covered by water in 4th simulation is much larger than those observed during 2nd simulation.
