# Supplementary material for: Exploring Solanum tuberosum Epoxide Hydrolase Internal Architecture by Water Molecules Tracking
Source: Biomolecules. 2018 Nov 12;8(4):143. doi: 10.3390/biom8040143 (PMC6315908; doi:10.3390/biom8040143)
Supplement: Supplementary file 1 [file biomolecules-08-00143-s001.zip › Table_S1.doc]

| **Variant** | **Stability effect** | **Activity effect** | **Selectivity effect** | **Ref** |
| --- | --- | --- | --- | --- |
| D105A | loss of stability | loss of activity | none | [1,6] |
| Y154F |  | loss of activity |  |  |
| Y235F |  | loss of activity |  |  |
| Y154F/Y235F |  | loss of activity |  |  |
| H300A or H300Q |  | loss of activity |  |  |
| H300N |  | some activity for R,R-TSO |  |  |
| E35Q/H300N | loss of stability | some activity for R,R-TSO | none | [6] |
| Y149F | loss of stability | decreased activity | decrease for R,R-TSO; comparable with WT for S,S-TSO | [2] |
| H153F |  |  |  |  |
| Y149F/H153F |  |  |  |  |
| K179Q | loss of stability | increased activity towards R-enantiomer | none | [3] |
| E215Q |  | increased activity towards R-enantiomer | loss of regioselectivity; very promiscuous |  |
| R236K or R236Q |  | increased activity towards S-enantiomer | higher discrimination favouring  C-1 attack |  |
| F33V or F33Y | no data | no data | none | [4] |
| W106F/L109I or W106F/L109V |  |  | higher discrimination favouring  S-enantiomer |  |
| V141K |  |  | lower discrimination favouring  S-enantiomer |  |
| V141K/I155V |  |  | higher discrimination favouring  R-enantiomer |  |
| W106L/L109Y/V141K/I155V |  |  |  |  |
| V141T or V141Q |  |  | lower discrimination favouring  S-enantiomer |  |
| V141T/L145I or V141T/L145T |  |  |  |  |
| I180L/F189C |  |  |  |  |
| I180V |  |  |  |  |
| F189I or F189V |  |  |  |  |
| V141Q/I155L |  |  |  |  |
| I180L |  |  | none |  |
| I180C/F189V |  |  | none |  |
| W106Y/L109I/I180F | no data | no data | shifted preference on attack on  C-1 in R-SO and on C-2 in  R,R-enantiomer of  *trans*-2-methylstyrene oxide | [5] |
| W106Y/L109I/L145R/I155Y |  |  |  |  |
| W106Y/L109I |  |  |  |  |
| V141K/I155V |  |  | shifted preference on attack on  C-2 in S-SO and on C-2 in  S,S-enantiomer of  *trans*-2-methylstyrene oxide | [5,7] |
| W106L/L109Y/V141K/I155V |  |  | shifted preference on attack on  C-1 in S-enantiomer  (2,3-epoxypropyl)benzene and on  C-2 in R,R-enantiomer of  *trans*-2-methylstyrene oxide | [5,7,8] |
| W106L/L109Y/I155V |  |  |  | [5] |
| W106L/L109Y/V141K/I155V/F189L |  |  |  | [5,7] |
| W106L/L109Y/V141K/I155V/F189L/L266F |  |  | shifted preference on attack on  C-1 in R-SO |  |
| W106L/L109Y/V141K/I155V/F189L/L266G |  |  | shifted preference on attack on  C-2 in R,R-enantiomer of  *trans*-2-methylstyrene oxide | [5] |

1. Elfström L.T., Widersten M. 2005 Catalysis of potato epoxide hydrolase, StEH1. Biochem J. 390:633-640.
2. Thomaeus A., Naworyta A., Mowbray S.L., Widersten M. 2008. Removal of distal protein–water hydrogen bonds in a plant epoxide hydrolase increases catalytic turnover but decreases thermostability. Protein Sci. 17(7): 1275–1284.
3. Lindberg D., Ahmad S., Widersten M. 2010. Mutations in salt-bridging residues at the interface of the core and lid domains of epoxide hydrolase StEH1 affect regioselectivity, protein stability and hysteresis. Archives of Biochemistry and Biophysics 495: 165–173.
4. Gurell, A., Widersten, M. 2010. Modification of Substrate Specificity Resulting in an Epoxide Hydrolase with Shifted Enantiopreference for (2,3-Epoxypropyl)benzene. ChemBioChem, 11(10), 1422–1429.
5. Carlsson Å.J., Bauer P., Ma H., Widersten M. 2012. Obtaining optical purity for product diols in enzyme-catalyzed epoxide hydrolysis: contributions from changes in both enantio- and regioselectivity. Biochemistry. 25;51(38):7627-37.
6. Amrein B.A., Bauer P., Duarte F., Janfalk Carlsson Å., Naworyta A., Mowbray S.L., Widersten M., Kamerlin S.C.L. 2015. Expanding the Catalytic Triad in Epoxide Hydrolases and Related Enzymes. ACS Catal., 5 (10): 5702–5713.
7. Janfalk Carlsson Å., Bauer P., Dobritzsch D., Nilsson M., Kamerlin S.C.L., Widersten M. 2016. Laboratory-Evolved Enzymes Provide Snapshots of the Development of Enantioconvergence in Enzyme-Catalyzed Epoxide Hydrolysis. ChemBioChem 17: 1693 – 1697.
8. Bauer P., Janfalk Carlsson Å., Amrein B. A., Dobritzsch D., Widersten M., Kamerlin S. C. L. 2016. Conformational diversity and enantioconvergence in potato epoxide hydrolase 1. Org Biomol Chem. 14(24): 5639–5651.
