# Supplementary material for: Exploring Solanum tuberosum Epoxide Hydrolase Internal Architecture by Water Molecules Tracking
Source: Biomolecules. 2018 Nov 12;8(4):143. doi: 10.3390/biom8040143 (PMC6315908; doi:10.3390/biom8040143)
Supplement: Supplementary file 1 [file biomolecules-08-00143-s001.zip › Table_S2.doc]

| ID | ALL | p(ALL) | IN | p(IN) | OUT | p(OUT) |
| --- | --- | --- | --- | --- | --- | --- |
| TM1 | 1893 ± 158 | 92.1 ± 7.7 | 940 ± 84 | 47.8 ± 4.1 | 954 ± 84 | 48.4 ± 4.1 |
| TC/M | 139 ± 28 | 6.8 ± 1.4 | 76 ± 9 | 3.9 ± 0.4 | 63 ± 10 | 3.2 ± 0.5 |
| TM2 | 22 ± 23 | 1.1 ± 1.1 | 11 ± 12 | 0.5 ± 0.6 | 11 ± 13 | 0.6 ± 0.7 |
| outliers | 2 ± 1 | < 0.1 | 1 ± 2 | < 0.1 | 1 ± 1 | < 0.1 |
