# Supplementary material for: Exploring Solanum tuberosum Epoxide Hydrolase Internal Architecture by Water Molecules Tracking
Source: Biomolecules. 2018 Nov 12;8(4):143. doi: 10.3390/biom8040143 (PMC6315908; doi:10.3390/biom8040143)
Supplement: Supplementary file 1 [file biomolecules-08-00143-s001.zip › Table_S3.doc]

| ID | IN-OUT | p(IN-OUT) | DIF | p(DIF) | N | p(N) |
| --- | --- | --- | --- | --- | --- | --- |
| TM1 | 858 ± 87 | 0.84 | 137 ± 32 | 0.13 | 41 ± 6 | 0.04 |
| TC/M | 9 ± 4 | 0.06 | 118 ± 16 | 0.91 | 3 ± 1 | 0.02 |
| TM2 | <1 ± 1 | 0.01 | 21 ± 24 | 0.97 | <1 ± 1 | 0.02 |
| outliers | 0 | 0.00 | 2 ± 1 | 0.75 | 1 ± 1 | 0.25 |
