# Supplementary material for: Exploring Solanum tuberosum Epoxide Hydrolase Internal Architecture by Water Molecules Tracking
Source: Biomolecules. 2018 Nov 12;8(4):143. doi: 10.3390/biom8040143 (PMC6315908; doi:10.3390/biom8040143)
Supplement: Supplementary file 1 [file biomolecules-08-00143-s001.zip › Table_S4.doc]

| Beginning | End | MD1 | MD2 | MD3 | MD4 | MD5 | SUM | [%] |
| --- | --- | --- | --- | --- | --- | --- | --- | --- |
| TM1 | TM1 | 797 | 931 | 750 | 907 | 904 | 4289 | 81.7 |
| TM1 | TC/M | 61 | 45 | 45 | 50 | 60 | 261 | 5.0 |
| TC/M | TM1 | 65 | 64 | 53 | 49 | 90 | 321 | 6.1 |
| TM1 | TM2 | 28 | 4 | 6 | 7 | 4 | 49 | 0.9 |
| TM2 | TM1 | 25 | 3 | 9 | 8 | 2 | 47 | 0.9 |
| TM1 | N | 23 | 19 | 14 | 18 | 23 | 97 | 1.8 |
| N | TM1 | 25 | 24 | 21 | 17 | 21 | 108 | 2.1 |
| TC/M | TC/M | 7 | 7 | 4 | 13 | 14 | 45 | 0.9 |
| TC/M | TM2 | 4 | 1 |  |  | 1 | 6 | 0.1 |
| TM2 | TC/M | 3 |  |  | 1 |  | 4 | 0.1 |
| TC/M | N | 1 | 2 | 3 |  | 3 | 9 | 0.2 |
| N | TC/M |  | 1 |  | 2 | 1 | 4 | 0.1 |
| TM2 | TM2 | 1 |  |  |  |  | 1 | <0.1 |
| TM2 | N | 1 |  |  |  |  | 1 | <0.1 |
| N | TM2 |  |  | 1 |  |  | 1 | <0.1 |
| TM1 | OUT | 1 |  | 1 | 1 |  | 3 | 0.1 |
| OUT | TM1 |  | 1 |  | 2 |  | 3 | 0.1 |
| N | N |  |  | 1 |  |  | 1 | <0.1 |
| OUT | N |  | 1 |  | 1 |  | 2 | <0.1 |
