# Supplementary material for: Exploring Solanum tuberosum Epoxide Hydrolase Internal Architecture by Water Molecules Tracking
Source: Biomolecules. 2018 Nov 12;8(4):143. doi: 10.3390/biom8040143 (PMC6315908; doi:10.3390/biom8040143)
Supplement: Supplementary file 1 [file biomolecules-08-00143-s001.zip › Table_S5.doc]

| 1^st^ MD simulation | | | | 2^nd^ MD simulation | | | | 3^rd^ MD simulation | | | | 4^th^ MD simulation | | | | 5^th^ MD simulation | | | |
| --- | --- | --- | --- | --- | --- | --- | --- | --- | --- | --- | --- | --- | --- | --- | --- | --- | --- | --- | --- |
| C ID | Occ [%] | AB [Å] | MB [Å] | C ID | Occ [%] | AB [Å] | MB [Å] | C ID | Occ [%] | AB [Å] | MB [Å] | C ID | Occ [%] | AB [Å] | MB [Å] | C ID | Occ [%] | AB [Å] | MB [Å] |
| 1 | 100 | 1.9 ± 0.4 | 3.1 | 1 | 100 | 2.0 ± 0.4 | 2.9 | 1 | 100 | 1.9 ± 0.4 | 2.9 | 1 | 100 | 1.9 ± 0.4 | 3.0 | 1 | 100 | 2.0 ± 0.4 | 3.0 |
| 2 | 86 | 1.7 ± 0.3 | 2.7 | 2 | 92 | 1.2 ± 0.2 | 1.9 | 2 | 92 | 1.7 ± 0.3 | 2.8 | 2 | 97 | 1.3 ± 0.2 | 2.0 | 2 | 85 | 1.2 ± 0.2 | 2.1 |
| 3 | 98 | 1.3 ± 0.2 | 2.1 | 3 | 69 | 1.3 ± 0.3 | 2.6 | 3 | 97 | 1.2 ± 0.2 | 2.0 | 3 | 70 | 1.3 ± 0.3 | 2.7 | 3 | 70 | 1.4 ± 0.3 | 2.7 |
| 4 | 65 | 1.3 ± 0.2 | 2.5 | 4 | 30 | 1.2 ± 0.2 | 2.0 | 4 | 47 | 1.2 ± 0.2 | 2.4 | 4 | 41 | 1.2 ± 0.2 | 2.0 | 4 | 30 | 1.1 ± 0.2 | 2.0 |
| 5 | 47 | 1.2 ± 0.2 | 2.5 | 5 | 27 | 1.0 ± 0.1 | 1.7 | 5 | 33 | 1.2 ± 0.2 | 2.6 | 6 | 24 | 1.0 ± 0.1 | 1.4 | 5 | 47 | 1.2 ± 0.2 | 2.5 |
| 6 | 21 | 1.0 ± 0.2 | 2.0 |  |  |  |  | 6 | 50 | 1.0 ± 0.1 | 1.4 | 7 | 23 | 1.0 ± 0.1 | 1.3 | 6 | 23 | 1.0 ± 0.1 | 1.5 |
| 7 | 28 | 1.0 ± 0.1 | 1.4 |  |  |  |  | 7 | 20 | 1.0 ± 0.1 | 1.8 | 8 | 21 | 1.0 ± 0.1 | 1.4 |  |  |  |  |
|  |  |  |  |  |  |  |  |  |  |  |  | 9 | 22 | 1.0 ± 0.1 | 1.4 |  |  |  |  |
|  |  |  |  |  |  |  |  |  |  |  |  | 10 | 20 | 1.0 ± 0.1 | 1.3 |  |  |  |  |
