# Supplementary material for: Exploring Solanum tuberosum Epoxide Hydrolase Internal Architecture by Water Molecules Tracking
Source: Biomolecules. 2018 Nov 12;8(4):143. doi: 10.3390/biom8040143 (PMC6315908; doi:10.3390/biom8040143)
Supplement: Supplementary file 1 [file biomolecules-08-00143-s001.zip › Table_S6.doc]

| MD | Entry | Exit | Tunnel ID (CAVER) | Residues at outliers’ exit | Outliers’ exit | No of events | Visited cavity | Total [ps] | Inp [ps] | Obj [ps] | Out [ps] |
| --- | --- | --- | --- | --- | --- | --- | --- | --- | --- | --- | --- |
| 1 | TM1 | O | 12 | F168, A169, V176, F228 | III | 1 | III | 636 | 27 | 273 | 330 |
| 2 | TM1 | O | 11 | Y190, A199 | II | 1 | III | 619 | 18 | 537 | 64 |
| 2 | O | TM1 | 8 | E165, F168, A169, G172, A173, E224 | III | 2 | III | 2206  2464 | 526  798 | 1138  958 | 542  708 |
| 2 | O | N | 14 | K178, T182, A203, V205, S208 | II | 1 | I | 27707 | 25485 | 2222 | N |
| 3 | TM1 | O | 16 | T182, R184, A203, P204, L207, S208 | II | 1 | I | 22547 | 1513 | 3439 | 17595 |
| 5 | O | TM1 | n.i. | R41, W210, L211, S212, E215, Q304 | I | 1 | - | 17423 | 15226 | 531 | 1664 |
| 5 | O | N | n.i. | R41, L211, S212, E215 | I | 1 | - | 19915 | 14666 | 5249 | N |
