# Supplementary material for: Exploring Solanum tuberosum Epoxide Hydrolase Internal Architecture by Water Molecules Tracking
Source: Biomolecules. 2018 Nov 12;8(4):143. doi: 10.3390/biom8040143 (PMC6315908; doi:10.3390/biom8040143)
Supplement: Supplementary file 1 [file biomolecules-08-00143-s001.zip › Table_S7.doc]

| Hot-spot localization |  | | | | |
| --- | --- | --- | --- | --- | --- |
|  | MD1 | MD2 | MD3 | MD4 | MD5 |
| TC/M entry | H153, **P188**, **L266**, **I270** | P186, A187, **P188**, **L266**, **I270** | **P188**, **L266**, **I270** | Y149, **P188**, **I270** | Y149, P186, A187, **P188**, H269, **I270** |
| TM2 entry |  | L116, F132, W248, A251 |  |  | R135, T249 |
| cavity I |  | T182, **Y183**, **R184**, **A299**, **H300***, **F301** | **Y183**, **R184**, D185, F264, L266, **A299**, **H300*** | **Y183**, **R184**, **A299**, **H300***, **F301** | I180, **Y183**, **R184**, D185, F189, F264, L266, **A299**, **H300***, **F301** |
| cavity II |  | F33, **E35***, D105*, H300*****, F301 |  | F33, **E35***, H300*****, F301 | **E35***, D105*, H230 |
| funnel interior | **L109**, **V130**, **H131**, **F132**, **S133**, **L238**, **N241**, **W242,** V267 | I78, **L109**, H113, **V130, H131**, **F132**, **S133**, N140, V141, **L238**, **N241**, **W242,** T245 | **L109**, **V130,** V141, **L238**, **N241**, **W242** | **L109**, **V130**, **H131,** **S133**, R135, **N241,**  T245, W248 | **L109**, **V130**, **H131,** **F132**, **S133** |
