# Supplementary material for: Exploring Solanum tuberosum Epoxide Hydrolase Internal Architecture by Water Molecules Tracking
Source: Biomolecules. 2018 Nov 12;8(4):143. doi: 10.3390/biom8040143 (PMC6315908; doi:10.3390/biom8040143)
Supplement: Supplementary file 1 [file biomolecules-08-00143-s001.zip › Table_S8.doc]

| Residue | Entropy score | Position of residue |
| --- | --- | --- |
| E35 | 0.28 | active site |
| L109 | 0.74 | funnel |
| V130 | 0.63 | funnel |
| H131 | 0.20 | funnel |
| F132 | 0.56 | funnel |
| S133 | 0.72 | funnel |
| Y183 | 0.79 | cavity I |
| R184 | 0.83 | cavity I |
| P188 | 0.68 | TC/M |
| L238 | 0.73 | funnel |
| N241 | 0.73 | funnel |
| W242 | 0.66 | funnel |
| L266 | 0.83 | TC/M |
| I270 | 0.85 | TC/M |
| A299 | 0.48 | cavity I |
| H300 | 0.13 | active site |
| F301 | 0.35 | cavity I |
